# Combustion Characteristics and Thermochemistry of Selected Silicon-Based Compositions for Time-Delay Detonators

**DOI:** 10.3390/ma18071456

**Published:** 2025-03-25

**Authors:** Marcin Gerlich, Waldemar A. Trzciński, Marcin Hara

**Affiliations:** 1 Faculty of Advanced Technologies and Chemistry, Military University of Technology, 00-908 Warsaw, Poland; marcin.gerlich@wat.edu.pl (M.G.); marcin.hara@wat.edu.pl (M.H.); 2 NITROERG S.A., 43-150 Bieruń, Poland

**Keywords:** pyrotechnic mixtures, pressure-independent combustion, adiabatic combustion temperature, X-ray diffraction, scanning electron microscopy, condensed-phase reactions

## Abstract

This study investigates the combustion characteristics of silicon-based time-delay compositions with bismuth(III) oxide (Bi_2_O_3_), antimony(III) oxide (Sb_2_O_3_), and lead(II,IV) oxide (Pb_3_O_4_) to identify formulations with pressure-independent burn rates. Unlike conventional pyrotechnic compositions, silicon-based mixtures offer an improved energy density and reduced sensitivity to pressure variations. The linear combustion rate of the compositions was determined for a wide range of silicon contents and for different compaction pressures. Experimental results show that burn rates range from 8 mm s⁻^1^ to 195 mm s⁻^1^, depending on the metal oxide type and silicon content. The highest rate (195 mm s⁻^1^) was observed for Si/Pb_3_O_4_ at 30 wt.% silicon, while Si/Sb_2_O_3_ had the lowest (10 ÷ 35 mm s⁻^1^). The calorimetric heat of combustion varied between 1200 J g⁻^1^ and 1400 J g⁻^1^, with adiabatic combustion temperatures reaching 2200 K, calculated from this heat. DTA and XRD confirmed the condensed-phase combustion, forming reduced metal phases and silicon oxides. SEM and EDS revealed a porous residue structure. This work introduces a novel approach to time-delay compositions using silicon as a primary fuel. It shows that specific silicon oxide–metal systems maintain stable combustion for different loading pressures and advance pyrotechnic formulations for safer and more efficient industrial and defense applications.

## 1. Introduction

The effectiveness and safety of the use of detonators in mining and military technology depends on the precise control and sequencing of detonation, a function governed mainly by the characteristics of pyrotechnic delay compositions. Traditional pyrotechnic compositions, which primarily produce gaseous products, have been pivotal in these applications. However, with evolving technological and safety standards, gasless pyrotechnic compositions are gaining prominence due to their stability and reliable performance under diverse conditions. The most important feature of the time-delay compositions currently used is the lack of influence (or little influence) of pressure on the burn rate of the mixtures. A constant burn rate is achieved by using compositions of which the components react with each other in a system of condensed bodies, i.e., at the solid–solid, solid–liquid, or liquid–liquid phase boundaries. The lack of thermal decomposition of the oxidant with subsequent oxidation of the fuel with gaseous oxygen implies that the rate of the oxidation reaction is not dependent on the pressure inside the detonator. There are, however, many other factors that can influence the burn rate (and consequently delay time of a detonator). The quantitative composition of a mixture is the most obvious one. From a technological point of view, it is important to know the dependence of the combustion rate on the mixture composition. This gives the possibility of selecting the fuel content in such a way that no significant changes in the rate of combustion are observed for a small change in the fuel content. Another important factor affecting the accuracy of detonators is the dependence of the burn rate of the composition on the density of the mixture. In practice, such compositions are used for which small variations in density do not significantly affect the rate of combustion.

Silicon is one of the most commonly used fuels in time-delay compositions. It is found in gasless pyrotechnic mixtures with metal oxides, such as Pb_3_O_4_, Sb_2_O_3_, Sb_6_O_13_, Bi_2_O_3_, SnO_2_, and Fe_2_O_3_, among others. In the present work, Sb_2_O_3_ and Bi_2_O_3_ were chosen for the study as representations of metal oxides in mixtures with Si that differ markedly in the rate of combustion [1]. On the other hand, Pb_3_O_4_, which is widely used in pyrotechnic mixtures, was chosen for comparative purposes.

The most common composition (used primarily in short-period time-delay detonators) is Si/Pb_3_O_4_. Silicon reacts with red lead (Pb_3_O_4_) in the condensed phase [2]. There are, however, also reactions taking place on solid–gas phase boundaries, making this composition combustion process not entirely pressure independent. The literature presents DTA curves of the Si/Pb_3_O_4_ mixture and proposes a combustion reaction mechanism [2,3,4]. Thermal analysis indicates that during the combustion of the Si/Pb_3_O_4_ mixture, Pb_3_O_4_ decomposes at around 540 °C with the release of two products: PbO and O_2_ [5]. The two products then react with Si to form SiO_2_. The burn rate of these mixtures is also subject to temperature variations. Experiments have shown that the burn rate of Si/Pb_3_O_4_ mixtures follows an exponential relationship with ambient temperature [4]. Interestingly, the temperature dependency of the burn rate is more pronounced in mixtures with higher silicon contents. This aligns with the hypothesis that a higher fuel content favors a solid–solid reaction mechanism, making the burning process more temperature-dependent due to the lower involvement of gaseous products.

Mixtures of silicon with Bi_2_O_3_ appear in the literature most often in the form of additions to already existing time-delay compositions [6,7]. Bismuth oxide also appears in the ternary mixture along with antimony oxide [8]. The linear combustion velocity of the Si/Bi_2_O_3_ mixture was determined for mixtures with a wide range of silicon contents from 5 to 40 wt.% [9]. Silicon powder with different grain sizes (from 0.91 to 3.94 µm) was used. It was shown that the linear combustion velocity in the system can range from about 10 mm/s to 160 mm/s (the velocity decreases with an increasing grain size). The range thus includes up to the combustion rate of the Si/Pb_3_O_4_ mixture, which explains the potential use of Bi_2_O_3_ as a Pb_3_O_4_ substitute. The effect of the tube material on the linear combustion velocity of the Si/Bi_2_O_3_ mixture was also determined in [9]. Using an aluminum tube reduced the combustion velocity by up to 40% compared to the velocities obtained in time-delay elements with lead tubes. Little is known about the combustion thermodynamics of the Si/Bi_2_O_3_ mixture—there is no information in the literature about the products of the combustion of this mixture, the heat of the reaction, and the adiabatic combustion temperature.

The properties of Si/Sb_2_O_3_ mixtures are better described in the literature. It is reported that the only the products of the combustion of this mixture are SiO_2_, antimony, and unreacted silicon (in the case of mixtures with a negative oxygen balance) [1,8]. The literature provides information on the combustion temperature of this mixture, which for mixtures containing from 30 to 50% silicon, it is approximately 1200 °C, and it is not subject to any biased changes within this silicon content range [10]. In addition to the results of the linear combustion rate, thermal analysis curves are also provided, indicating that the first exothermic effect occurs at approximately 600 °C [10].

The present work is a continuation of the research on gasless pyrotechnic mixtures carried out in papers [11,12] to develop advanced time-delay detonators. The study in this work focuses on the combustion process of silicon-based mixtures with bismuth(III) oxide (Bi_2_O_3_), antimony(III) oxide (Sb_2_O_3_), and lead(II,IV) oxide (Pb_3_O_4_). The main goal is to find compositions that exhibit burn rates that are independent of the pressure inside the detonator, thus ensuring the reliability and consistency of its performance. Therefore, the burn rates of Si/Sb_2_O_3_, Si/Bi_2_O_3_ and Si/Pb_3_O_4_ mixtures were systematically studied by changing the composition of the mixtures and the loading density of the samples. To validate the reaction mechanism between silicon and metal oxides, a thermal analysis was conducted. In addition to X-ray diffraction of the solid combustion products, calorimetry and the calculation of the adiabatic temperature were employed. These methods collectively enhance the understanding of the combustion behavior of silicon-based compositions. The results obtained expand knowledge of the combustion characteristics of the mixtures and the compositions and structures of their combustion products.

## 2. Materials and Methods

### 2.1. Materials

**Figure 1:**
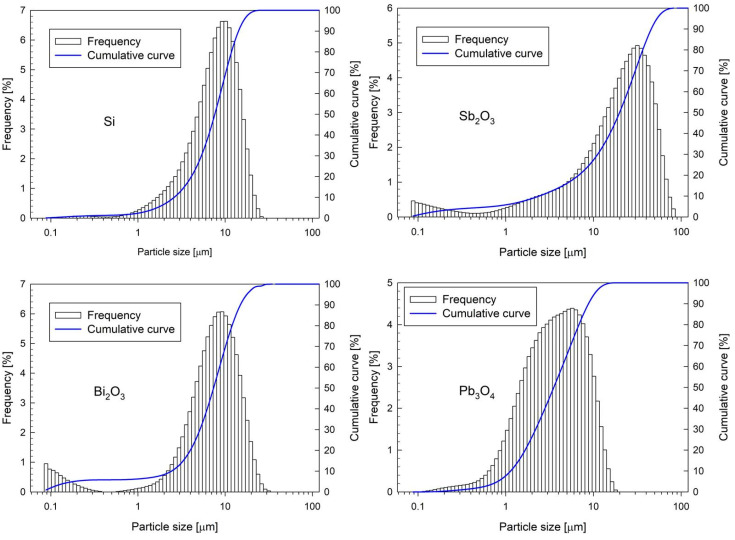
Particle size distribution of Si, Sb_2_O_3_, Bi_2_O_3_, and Pb_3_O_4_.

Micron-sized silicon, Sb_2_O_3_, Bi_2_O_3_, and Pb_3_O_4_ were used. The purity of all powders was over 99%. The grain size analysis of the powders was performed using an ANALYSETTE 22 MicroTec plus laser particle size analyzer (FRITSCH GmbH, Idar-Oberstein, Germany). Figure 1 presents the grain size distribution of the applied components.

All components used are characterized in Table 1. In addition to the theoretical maximum density (*TMD*) of the components, the volume-weighted mean diameter (De Brouckere *D*_[4,3]_ diameter) and volume-weighted *D*_99_ diameter are presented in the table. All powders are characterized by micrometric particle sizes. The largest grain size was antimony oxide, of which the mean diameter *D*_[4,3]_ is 23.8 microns. For the remaining components, the average particle diameter is less than 10 microns. Nanometer-sized particles are also seen for Sb_2_O_3_ and Bi_2_O_3_ in Figure 1.

**Figure 2:**
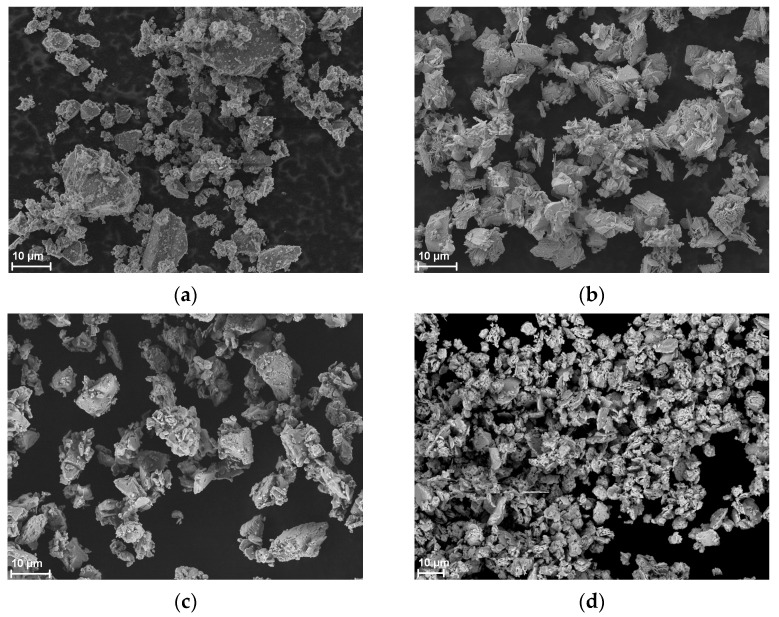
SEM images of powder (**a**) Si, (**b**) Si/Sb_2_O_3_, (**c**) Bi_2_O_3_, and (**d**) Pb_3_O_4_.

Figure 2 shows SEM images of the powders used. SEM images reveal that the particles of all materials exhibit irregular shapes. In fact, they are conglomerates of smaller particles. When examining the grain distribution with the technique used, they can break into smaller particles, hence the appearance of a large number of nanometer-sized particles in the mass distribution in the case of Sb_2_O_3_ and Bi_2_O_3_.

Generally, the use of nanometer particles is not justified when pyrotechnic mixtures are used as time-delay mixtures. To prevent fragmentation of the mixture inside the time-delay element, high working pressures of up to 400 MPa are used. The literature reports that at such high loading pressures (resulting in a significant increase in the density of the mixture), the burn rate of mixtures based on nanometric components is similar to those with micrometric components [1,13,14]. Therefore, it is not possible to significantly affect the linear combustion rate of the mixture through the presence of small amounts of fine-grained components.

### 2.2. Sample Preparation

Delay elements were produced using tubes made of a zinc–aluminum (ZnAl) alloy. The tubes, manufactured via die-casting, had dimensions of 26 mm in length, with an inner diameter of 3 mm and an outer diameter of 6 mm. Before use, all tubes were verified for dimensional accuracy and cleaned of residual machine oil from the casting process. To minimize friction during the pressing of the pyrotechnic composition, a thin graphite coating was applied to the inner walls.

Si/Sb_2_O_3_, Si/Bi_2_O_3_, and Si/Pb_3_O_4_ mixtures with silicon contents from 5 wt.% to 60 wt.% were prepared. Approximately 100 g of each composition was blended for 6 h using a TURBULA dry mixer. As the raw materials were non-hygroscopic, pre-drying was unnecessary.

The formation of time-delay components involved the multi-step pressing of small, volumetrically measured doses of the selected composition. This method ensured that weight deviations remained within 2 wt.%. A hydraulic press equipped with a strain gauge for pressure monitoring was used. Samples were compacted at pressures of 200, 300, and 400 MPa, with the pyrotechnic column in each element measuring 20 mm in height. The number of applied doses varied between 10 and 12, depending on the composition and applied pressure.

**Figure 3:**
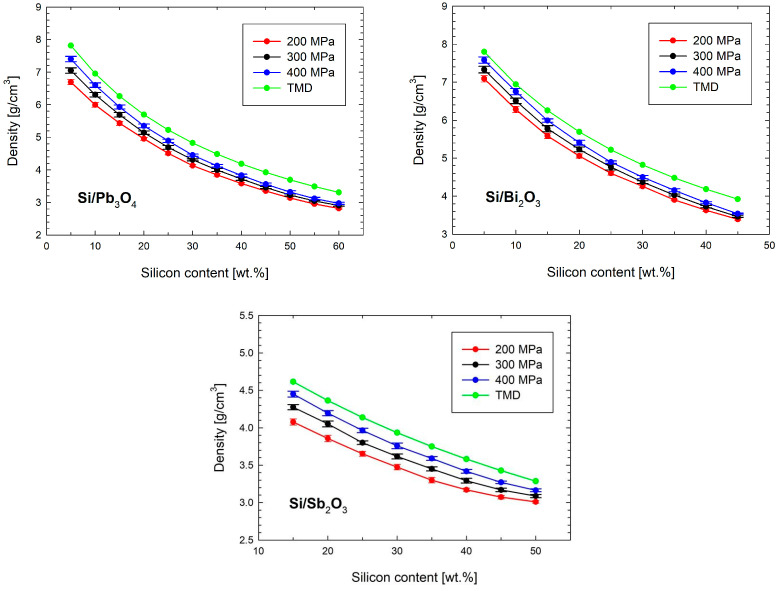
Density of tested mixtures as a function of the silicon content for different loading pressures.

To verify the loading precision, each ZnAl tube was weighed before and after filling with the mixture. The density of the compacted composition was calculated based on the mass and volume of the mixture within the tube. Figure 3 illustrates the relationship between the density and silicon content.

As expected, increasing the silicon content led to a notable reduction in density. Raising the compaction pressure from 200 to 400 MPa resulted in density increases of 5–10 wt.% for mixtures with Pb_3_O_4_, and 5–9 wt.% for those containing Bi_2_O_3_ and Sb_2_O_3_.

### 2.3. Burn Rate Measurements

**Figure 4:**
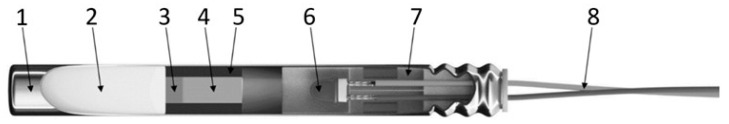
Schematic of an electric detonator: 1—shell, 2—secondary charge, 3—primary charge, 4—time-delay composition, 5—ZnAl tube, 6—fuse head, 7—shielding plug, 8—wire [12].

The propagation speed of the combustion front was evaluated by measuring the delay time in electric detonators containing the tested compositions. A schematic of the detonator is provided in Figure 4 [12]. The mean linear burn rate was determined based on the known pressed composition length and recorded combustion duration.

The tests were conducted using detonators with steel wiring and aluminum casings. The initiating charge consisted of lead azide (Pb(N_3_)_2_), while the secondary charge comprised pressed pentaerythritol tetranitrate (PETN). The energy output of the pyrotechnic fuse head was estimated at 0.12 kJ.

Delay times were recorded using an ohmmeter–microphone system. Timing commenced upon the application of an electrical pulse and concluded upon detecting the detonator’s explosion via a microphone. Measurements were taken with an accuracy of 0.1 ms. Since the microphone was positioned 2 m from the detonator, recorded times were corrected for the travel time of the shock wave. Additionally, the average delay time of the fuse head was subtracted from the total delay time of the detonator.

### 2.4. Heat of Combustion

The heat of combustion was determined for unpressed samples weighing approximately 15 g, which were placed in 4 mL quartz crucibles. A KL-12 calorimeter was employed for the measurements, utilizing a 350 mL steel combustion chamber submerged in a 2750 mL steel vessel filled with distilled water. The ignition system included a thin Kanthal D alloy resistance wire. All tests were conducted under an argon atmosphere at 20 MPa. To ensure oxygen exclusion, the calorimetric bomb was purged with argon for 120 s before ignition.

### 2.5. Differential Thermal Analysis

Thermal analysis of Si/Sb_2_O_3_ and Si/Bi_2_O_3_ mixtures containing 20 wt.% silicon was carried out using a Labsys thermal analyzer (Setaram Instrumatation, Caluire, France). To maintain an inert atmosphere, a Bronkhorst mass flow controller was integrated into the analyzer, allowing for a precise adjustment of gas flow. Each test used approximately 3 mg of the sample, placed in an Al_2_O_3_ crucible with a 100 µL capacity. The argon flow rate was maintained at 50 mL min⁻^1^, and the sample was heated at 10 °C min⁻^1^ up to 1100 °C.

### 2.6. Combustion Product Analysis

The qualitative composition of solid combustion products was analyzed using X-ray diffraction (XRD) with a SmartLab 3 kW diffractometer (Rigaku Polska, Wrocław, Polska). The system utilized a Cu lamp (40 kV, 30 mA) and a 1D Dtex250 linear detector. Data interpretation was performed with the ICDD PDF4+2022 database and the PDXL analytical program. The measurements followed the Bragg–Brentano (BB) theta-2theta geometry within a 2θ range of 10–90°, using a 0.02° step size and a traverse speed of 2° min⁻^1^. Powder samples were placed on a “backgroundless” Si510 single-crystal stage for analysis.

A CrossBeam 540 scanning electron microscope with a FE- SEM field emission cathode (Carl Zeiss, Jena, Germany) was used to observe the morphology of the components and reaction products. The resolution in SEM mode was 0.9 nm. The electron microscope was equipped with intra-lens detectors to provide the desired imaging contrast. An EDS (Energy Dispersive Spectroscopy) detector with an area of 80 mm^2^ was used for elemental analysis. The use of a detector with such an area made it possible to quickly perform an elemental analysis of the surface of combustion reaction products. The microscope was equipped with a Focused Ion Beam (FIB) system allowing cross sections (lamellas), as well as, in conjunction with the SEM column, reconstructions of the internal structure and elemental composition of materials with nanometer resolution. The microscope chamber contained three independently controlled micromanipulators allowing the transfer of material to supporting substrates.

### 2.7. Calculation of Heat of Combustion and Combustion Temperature

The theoretical heats of combustion were calculated for the assumed combustion reactions of the mixtures with silicon, with the assumption that all reaction products are in the condensed state. The heat of reaction was calculated using the following expression [12]:

(1) $${Q_{r}=-∆H}_{r}=-\left[\sum_{i=1}^{k}n_{i}{\left(\Delta H_{fT_{0}}^{0}\right)}_{i}-\sum_{j=1}^{l}n_{j}{\left(\Delta H_{fT_{0}}^{0}\right)}_{j}\;,\right]$$

where ${\left({\Delta H}_{fT0}^{0}\right)}_{i}$ is the enthalpy of formation of the *i*-th combustion product at the standard condition (*p*_0_ = 1 atm, *T*_0_ = 298.15 K), *n_i_*—the number of moles of the *i*-th combustion product, *k*—the number of combustion products, ${\left({\Delta H}_{fT0}^{0}\right)}_{j}$—the enthalpy of formation of the *j*-th substrate of the composition, *n*_j_—moles of *j* component, *l*—number of pyrotechnic components. The values of enthalpies of formation for the components of the mixtures studied and their products of combustion were taken from the JANAF tables [15].

The calculation of the adiabatic combustion temperature is possible using the measured heat of combustion and the assumption of the composition of the combustion products. The method involves determining the amount of heat required to raise the temperature of the combustion products of the pyrotechnic mixture to a given temperature, *T_a_*, and comparing it with the experimentally measured heat [12]:

(2) $$Q_{exp}=\sum_{i=1}^{n}x_{i}\left\{\int_{T_{0}}^{T_{m,i}}{\left[C_{ps}\left(T\right)\right]}_{i}dT+{\left(Q_{m}\right)}_{i}+\int_{T_{m,i}}^{T_{a}}{\left[C_{pl}\left(T\right)\right]}_{i}dT\right\}\;\;,$$

where ${\left[C_{p}\left(T\right)\right]}_{i}$ is the specific heat of the *i*-th combustion product at the temperature *T* (the subscript *s* refers to solid and *l* to liquid), *x_i_* is the mole fraction of the *i*-th product, *T_m,i_* and (Q*_m_*)*_i_* are the melting temperature and the melting heat of the *i*- th product, respectively, *n*—the number of combustion products, *T*_0_ = 298.15 K.

In order to be able to perform the integration in Formula (2), the polynomial approximation of the dependence of specific heat on the temperature in the form of a table was carried out for the components of mixtures of silicon with metal oxides and their combustion products, using data from tables [15,16]. The following form of the approximating function for the individual components was adopted:

(3) $$C_{p}=C_{1}+C_{2}\theta +C_{3}\theta^{2}+C_{4}\theta^{3}+C_{5}\theta^{-2}\;,$$

where C_1_, C_2_, …, C_5_ are constant coefficients, *θ* = *T*/1000, *T*—absolute temperature. For the simpler *C_p_*(*T*) relationship, a linear function was used.

## 3. Results and Discussion

### 3.1. Burn Rate

**Figure 5:**
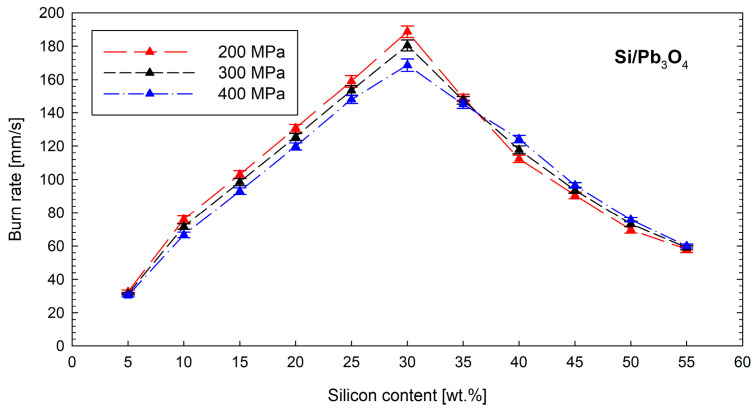
Influence of the silicon content and loading pressure on the burn rate of Si/Pb_3_O_4_ mixture.

The research on the burn rate of silicon-based compositions began with a mixture commonly used in short-period detonators, i.e., the Si/Pb_3_O_4_ composition. Five tests were performed for each composition and loading pressure. Figure 5 shows the linear burn rate of this mixture as a function of fuel content and loading pressure along with the standard deviation of the measured burn rates. The Si/Pb_3_O_4_ mixture is characterized by a wide range of linear burn rates (from approximately 30 to 190 mm s^−1^). In the time-delay element, stable propagation of the combustion front occurred for mixtures containing between 5 and 55 wt.% of silicon. The composition with a positive oxygen balance (containing 5 wt.% silicon) exhibited the lowest burn rate. The maximum linear burn rate is observed for a mixture containing 30 wt.% silicon. One of the most important and unexpected relationships shown in Figure 5 is the influence of the loading pressure on the value of the linear burn rate. For mixtures containing 30 wt.% or less silicon, an increase in the loading pressure results in a decrease in the linear burn rate. Such a relationship is typical for pyrotechnic mixtures, the main energy effect of which comes from the reaction occurring at the solid–gas interface. Interestingly, the burn rate of mixtures with higher silicon contents increased as the loading pressure rose. Such a relationship is characteristic of pyrotechnic mixtures, in which the solid–solid reaction dominates [1]. The change in the dependence of the combustion rate on the loading pressure (and consequently the density of the mixture) observed in Figure 3 indicates that the combustion of the Si/Pb_3_O_4_ mixture is a complex process in which at least two parallel exothermic reactions take place. One of them (dominant for mixtures with silicon content below 30 wt.%) is based on the oxidation of fuel with oxygen coming from the thermal decomposition of the oxidant. An increased combustion rate due to the increase in the mixture density for compositions containing more than 30 wt.% silicon indicates that the second reaction must be a reaction taking place in a system of condensed phases. The literature leaves no doubt on this issue, proposing the following reaction:

(4) $$\mathrm{Si}+\mathrm{PbO}\to \;{\mathrm{SiO}}_{2}+\mathrm{Pb}$$

**Figure 6:**
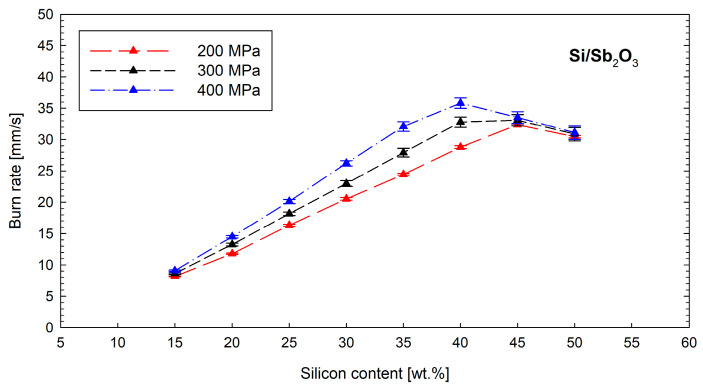
Influence of silicon content and loading pressure on the burn rate of the Si/Sb_2_O_3_ mixture.

Tests on the Si/Sb_2_O_3_ mixture showed that the combustion front can propagate in time-delay elements for mixtures containing 15 to 50 wt.% silicon. The assumption of the following combustion reaction,

(5) $$3\;\mathrm{Si}+2\;{\mathrm{Sb}}_{2}O_{3}\to \;3\;{\mathrm{SiO}}_{2}+4\;\mathrm{Sb}$$

implies that the stoichiometric silicon content is 12.6 wt.%. Therefore, silicon and antimony oxide mixtures with a positive oxygen balance have insufficient heat of reaction to sustain a stable combustion front. Figure 6 shows the variation in the linear combustion rate of the tested mixture as a function of the silicon content and loading pressure.

The Si/Sb_2_O_3_ mixture exhibits significantly lower combustion rates compared to its counterpart containing Pb_3_O_4_ (from about 8 mm s^−1^ for a mixture containing 15 wt.% silicon to about 37 mm s^−1^ for a fuel content of 40 wt.%). For this mixture, an increase in the burn rate with an increasing loading pressure is observed across the entire range of fuel contents, suggesting that the reactions in the system are likely solid–solid reactions. Interestingly, the smallest dependence of the burn rate on the loading pressure is observed for mixtures containing borderline silicon contents—i.e., 15 and 50 wt.%. For these mixtures, there is no significant difference in the burn rate between the time-delay compositions pressed at 200 MPa and 400 MPa.

**Figure 7:**
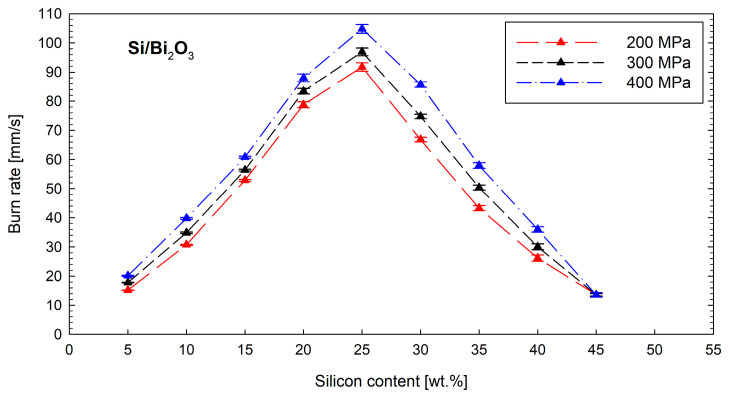
Influence of the silicon content and loading pressure on the burn rate of the Si/Sb_2_O_3_ mixture.

The linear combustion rate of the Si/Bi_2_O_3_ mixture is shown in Figure 7. Test results showed that the mixture burns stably for silicon contents in the range of 5 to 45 wt.%. Similarly, as in the case of the Sb_2_O_3_ mixture, an increase in the combustion rate with an increasing elaboration pressure is observed for mixtures with the same silicon content. This indicates a gasless combustion mechanism. The lowest combustion rate is for a mixture with an extremely negative oxygen balance, i.e., for a mixture containing 45 wt. % silicon. However, the combustion rate of Si/Bi_2_O_3_ reaches almost 110 mm s^−1^, raising the prospect of using it as a replacement for the mixture containing red lead. On the other hand, during tests with the Si/Bi_2_O_3_ mixture, detonators with significantly shorter delay times were obtained compared to detonators based on the Si/Sb_2_O_3_ mixture.

### 3.2. Thermal Analysis

**Figure 8:**
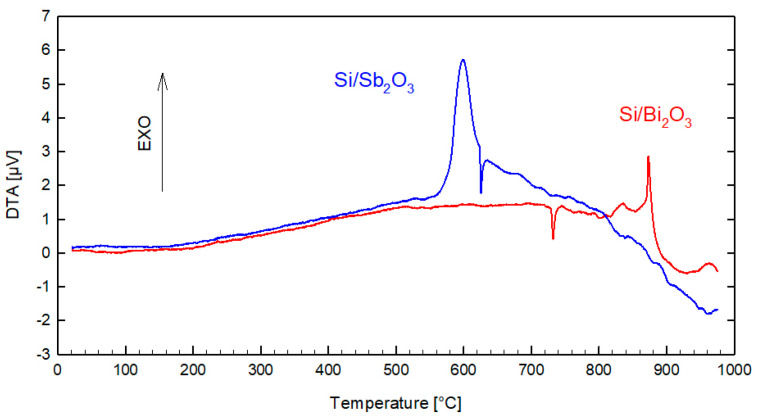
Thermograms for Si/Sb_2_O_3_ and Si/Bi_2_O_3_ mixtures with 20 wt.% silicon (temperature rate 10 K min^−1^, argon 50 mL min^−1^).

Figure 8 shows DTA curves for Si/Sb_2_O_3_ and Si/Bi_2_O_3_ mixtures, each containing 20 wt.% silicon. The DTA curve for the Si/Sb_2_O_3_ mixture shows an exothermic effect starting at about 560 °C, with a maximum at 600 °C. At approximately 630 °C, an overlapping endothermic peak emerges, partially coinciding with the exothermic effect. It is reported in [10] that for a mixture with a 40 wt.% Si content, this effect is seen in the temperature range of 500 ÷ 540 °C. The literature reports that Sb_2_O_3_ undergoes a solid–liquid phase transition at 655 °C and turns into a gas at 1456 °C (dimerizing to Sb_4_O_6_) [17]. The exothermic effect seen in the thermogram is therefore not a thermal decomposition of the oxidant. Silicon at a normal pressure up to 1414 °C also does not undergo polymorphic or phase transformations. The apparent exothermic effect must therefore come from the reaction of the components at the solid–solid interface, confirming the gasless nature of the reaction in the Si/Sb_2_O_3_ mixture.

There are two important energetic effects in the DTA curve of the Si/Bi_2_O_3_ mixture. The first, endothermic, begins at about 730 °C. This effect should be attributed to the polymorphic transformation of α-Bi_2_O_3_ into β-Bi_2_O_3_ [18]. The second effect is the exothermic effect occurring at 820 °C, which should be considered a reaction between two components. The melting point of Bi_2_O_3_ is 817 °C. Thus, the exothermic effect on the DTA curve for the Bi_2_O_3_/Si mixture appears at a temperature corresponding to the melting point of Bi_2_O_3_. Therefore, it can be considered that the reaction between silicon and bismuth oxide occurs in the solid–liquid interface.

### 3.3. Combustion Product Analysis

To theoretically estimate the heat of combustion and adiabatic combustion temperature, it is necessary to know the qualitative and quantitative composition of the combustion products. Thus, the composition of the solid products of the tested mixtures obtained after measuring the heat of reaction in a calorimetric vessel filled with argon was investigated. The initial powdered samples were solid samples in various forms after the reaction, meaning that some products were in the liquid phase during combustion. For the Si/Sb_2_O_3_ mixture with 15 wt.% silicon, the sample was compact and glassy after burning, and for 20 and 30 wt.% Si, the samples were compact and porous, while for 40 and 50 wt.%, the samples were spongy and swollen. The Si/Bi_2_O_3_ combustion product for 5 and 10 wt.% Si contents was compact and glassy, while for other Si contents, the samples were porous and spongy. The samples produced from burning the Si/Pb_3_O_4_ mixture were different. For 5 and 10 wt.% Si, the products were compact and porous, but above 15 wt.% Si, the samples were powdery, and their volume increased with a higher silicon content. The samples were crushed and subjected to XRD and SEM-EDS analysis.

**Figure 9:**
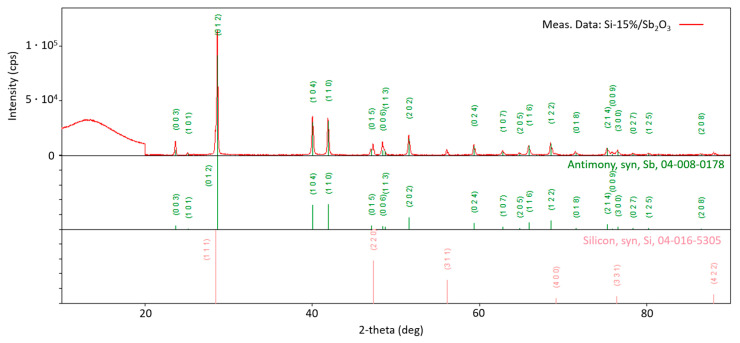
Diffractogram of the combustion products of the Si/Sb_2_O_3_ mixture containing 15 wt.% Si.

Since the products of the tested mixtures occur almost exclusively (except for trace amounts of sublimated compounds) in the solid phase, the qualitative composition can be determined via X-ray diffraction analysis. Figure 9 shows the diffractogram of the combustion products of the Si/Sb_2_O_3_ mixture (15 wt.% Si). The identified phases are metallic antimony and metallic silicon (representing unreacted fuel due to the negative oxygen balance of the mixture). The presence of metallic antimony is understood from reaction (5). The diffractogram shows a blurring of reflections for an angle of 2θ below 20 degrees. This is due to the presence of an amorphous phase in the sample, which is most likely SiO_2_. This conclusion seems reasonable based on the lack of identification of any compounds containing oxygen atoms. Therefore, it should be concluded that the entire main product of reaction (5) is amorphous SiO_2_.

**Figure 10:**
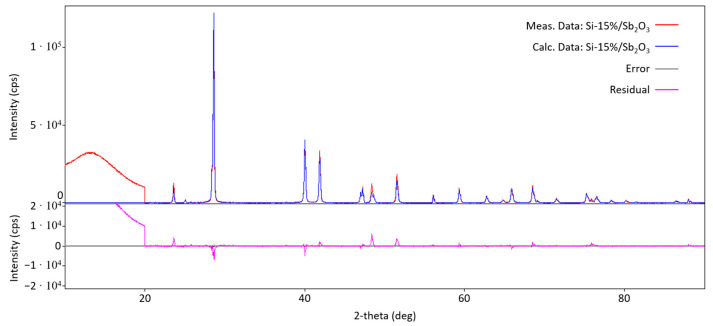
Comparison of the diffractogram obtained for the Si/Sb_2_O_3_ (15 wt.% Si) products with the curve determined theoretically for the identified products.

In order to determine the accuracy of the method, an X-ray pattern was theoretically determined for the obtained composition of combustion products and compared with experimental data (Figure 10). The degree of coverage of reflections in the diffractograms is satisfactory, which gives grounds to claim that correct conclusions have been drawn regarding the qualitative composition.

To determine whether changing the fuel content affects the composition of the reaction products, diffractograms of the combustion products of a Si/Sb_2_O_3_ mixture containing 30 wt.% Si were also taken. They showed no change in the qualitative composition of the combustion products compared to the mixture containing 15 wt.% Si.

**Figure 11:**
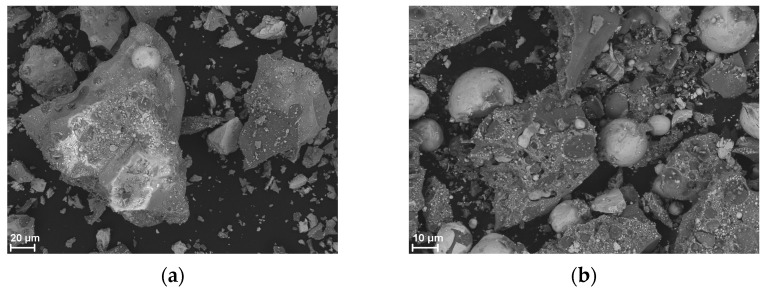
SEM images of Si/Sb_2_O_3_ combustion products for mixtures containing 15 wt. Si (**a**) and 30 wt. Si (**b**).

**Figure 12:**
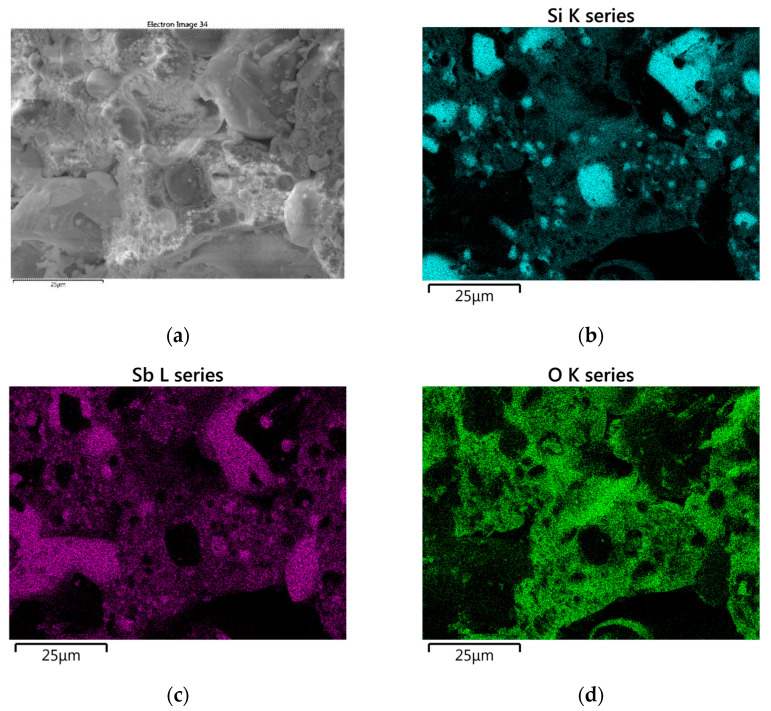
SEM-EDS elemental mapping of Si/Sb_2_O_3_ combustion products for a mixture containing 30 wt. Si (SEM image (**a**) and analogous elemental mapping of the element Si (**b**), Sb (**c**), O (**d**)).

Figure 11 shows SEM images of the solid combustion products of the Si/Sb_2_O_3_ mixture (15 and 30 wt.% Si), and Figure 12 shows the results of the SEM-EDS elemental mapping of the products (30 wt.% Si). The SEM images in Figure 11 show bright spherical antimony inclusions, as confirmed by the mapping results in Figure 12. Silicon particles are also present.

The usual reaction for the Si/Bi_2_O_3_ mixture is taken as follows:

(6) $$3\;\mathrm{Si}+2\;{\mathrm{Bi}}_{2}O_{3}\to \;3\;{\mathrm{SiO}}_{2}+4\;\mathrm{Bi}$$

This reaction corresponds to a stoichiometric silicon content of 8.27% by mass.

**Figure 13:**
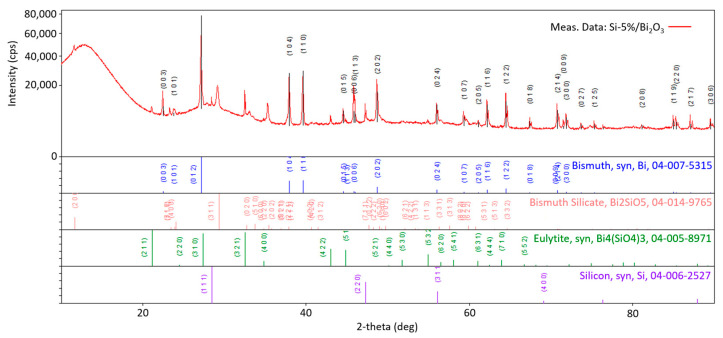
Diffractogram of the combustion products of the Si/Bi_2_O_3_ mixture containing 5 wt.% Si.

Interesting results were obtained for the combustion products of the Si/Bi_2_O_3_ mixture containing 5 wt.% of silicon, i.e., for a mixture with a positive oxygen balance. Figure 13 shows the diffractogram of the combustion products obtain for this mixture.

For the combustion products of the Si/Bi_2_O_3_ composition containing 5 wt.% Si, constructive interference of X-ray radiation was recorded for angles characteristic not only for bismuth and silicon, but also for bismuth and silicon oxides: Bi_2_SiO_5_ and Bi_4_(SiO_4_)_3_. These silicates are evidence of the existence of secondary reactions between the primary combustion product (amorphous SiO_2_) and the substrate in the form of bismuth oxide. These silicates are formed according to the following reaction:

(7) $${\mathrm{SiO}}_{2}+{\mathrm{Bi}}_{2}O_{3}\to \;{\mathrm{Bi}}_{2}{\mathrm{SiO}}_{5}$$

(8) $$3{\mathrm{SiO}}_{2}+{2\mathrm{Bi}}_{2}O_{3}\to \;{\mathrm{Bi}}_{4}{({\mathrm{SiO}}_{4})}_{3}$$

It is reported that the reaction between SiO_2_ and Bi_2_O_5_ takes place at a temperature of 625 °C [19]. It should be noted, however, that at this temperature there is no substrate for this reaction in the form of SiO_2_ in the system yet. Therefore, this reaction can only take place after the mixture reaches a temperature of 820 °C and follows the main reaction of Si with Bi_2_O_3_.

A diffraction pattern was also made for the mixture containing a larger amount of fuel (30 wt.% Si). In the case of a mixture with a strongly negative oxygen balance, no reflections from bismuth silicate were recorded. The reason for this is the lack of Bi_2_O_3_ in the mixture at the time of SiO_2_ formation—the entire oxidant reacts with the fuel grains. Therefore, the only compounds identified via XRD are bismuth and unreacted silicon.

**Figure 14:**
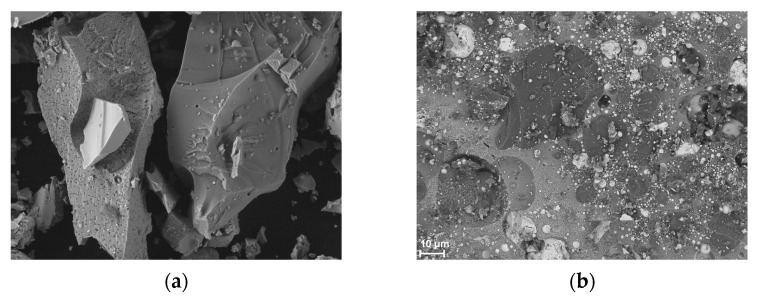
SEM images of Si/Bi_2_O_3_ combustion products for mixtures containing 5 wt. Si (**a**) and 30 wt. Si (**b**).

**Figure 15:**
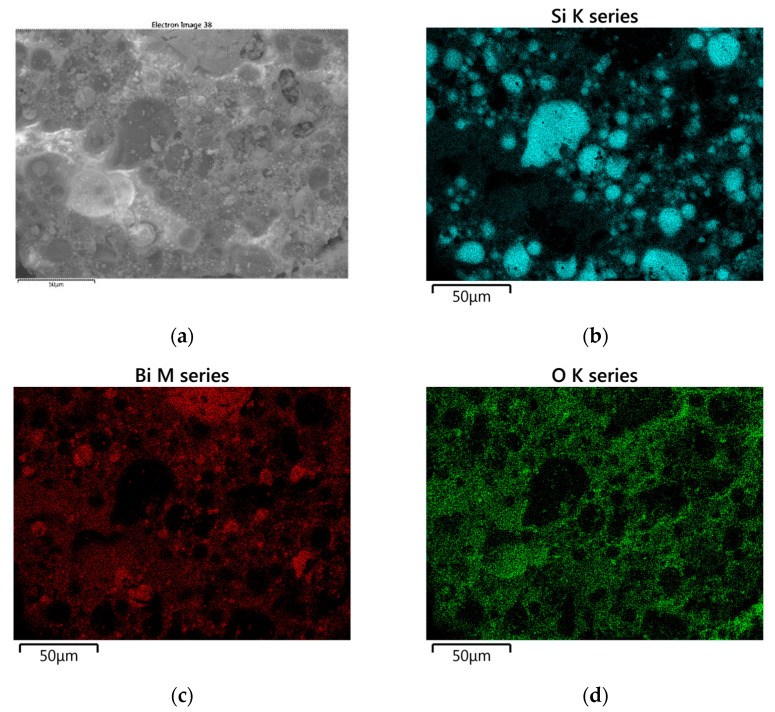
SEM-EDS elemental mapping of Si/Bi_2_O_3_ combustion products for a mixture containing 20 wt. Si (SEM image (**a**) and analogous elemental mapping of the element Si (**b**), Bi (**c**), O (**d**)).

Figure 14 shows SEM images of the solid combustion products of the Si/Bi_2_O_3_ mixture (5 and 30 wt.% Si), and Figure 15 shows the results of SEM-EDS elemental mapping of the products (20 wt.% Si). SEM-EDS images confirm the presence of unreacted silicon particles in the combustion products.

**Figure 16:**
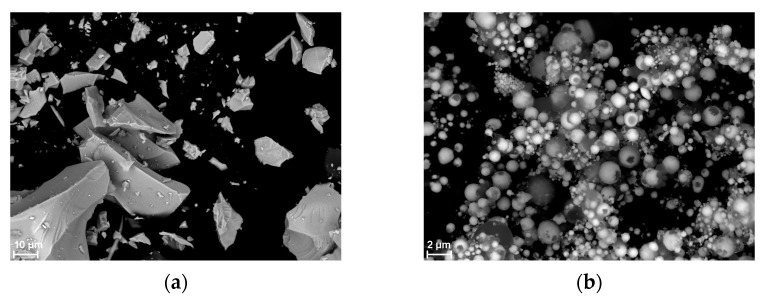
SEM images of Si/Pb_3_O_4_ combustion products for mixtures containing 5 wt. Si (**a**) and 10 wt. Si (**b**).

**Figure 17:**
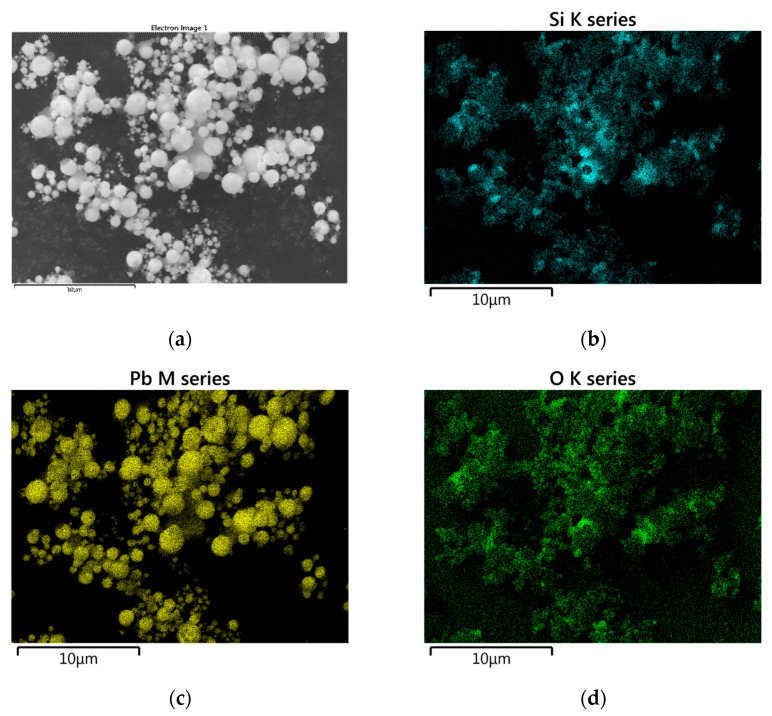
SEM-EDS elemental mapping of Si/Pb_3_O_4_ combustion products for a mixture containing 10 wt. Si (SEM image (**a**) and analogous elemental mapping of the element Si (**b**), Pb (**c**), O (**d**)).

No XRD analysis was performed for the combustion products of the Si/Pb_3_O_4_ mixture. SEM images of samples after the combustion of this mixture with 5 and 30 wt.% Si are shown in Figure 16. The sample in Figure 16b was in powder form. The results of SEM-EDS analysis for a mixture with 30 wt.% Si are shown in Figure 17. Both the SEM and EDS images distinguish lead in the form of spherical particles and amorphous SiO_2_.

### 3.4. Heat and Temperature of Combustion

**Figure 18:**
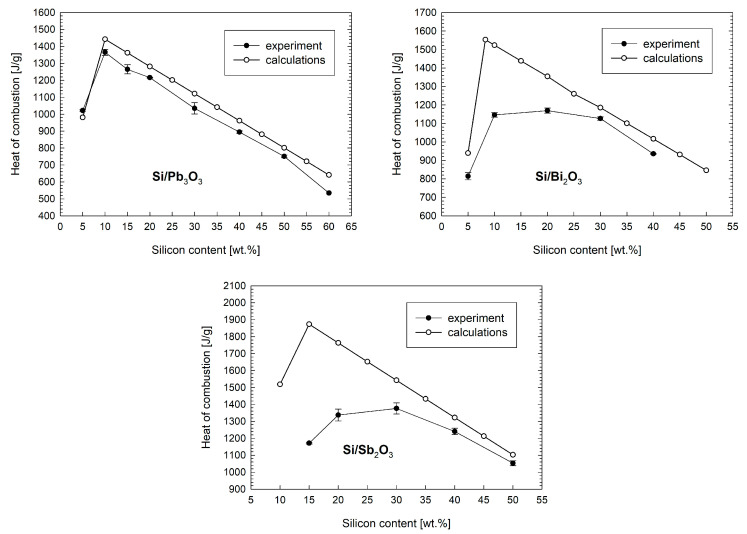
Calorimetric and calculated heats of combustion of tested mixtures as a function of the silicon content.

Calorimetric heats of combustion were measured two times for each composition of the pyrotechnic mixture. The average heat values with maximum deviation from the mean for the tested mixtures with Si are shown in Figure 18. The theoretical heat of combustion calculated according to the methodology described in Section 2.7 is also included in the figure. The maximum theoretical heat of combustion is obtained for mixtures with the stoichiometric content of components, assuming that the combustion product is SiO_2_.

The experimental results of the heat of combustion of the Si/Pb_3_O_4_ mixture are most consistent with the results of theoretical calculations. The maximum value of the heat of combustion for this mixture was measured for an Si content close to stoichiometric. Starting from a content of 10 wt.% Si, the measured heats of combustion are lower, within 50–100 J g^−1^, than the theoretical ones. A similar relationship was found in the paper [3], and the heats of combustion measured there for mixtures containing Si with a particle size of 5 μm are comparable to the values shown in Figure 18.

For Si/Bi_2_O_3_ and Si/Sb_2_O_3_ mixtures, the maximum heat of combustion was measured for Si contents much higher than stoichiometric. The measured heat of combustion of the Si/Sb_2_O_3_ mixture as a function of the Si content correlates well with the calorimetric heat of combustion of this mixture reported in the paper [10] despite the use of different Si fractions. A noticeable feature of the relationships in Figure 18 for high Si contents is the convergence of the combustion heat curve determined empirically with the one determined theoretically. It is more pronounced for the Si/Sb_2_O_3_ mixture. The difference between the curves decreases with an increasing silicon content. The authors suspect that this is due to the strong control of the combustion process by diffusion. The lower the fuel content, the more likely the oxidant grains are to contact each other and the more difficult it is for ions to penetrate the SiO_2_ combustion product barrier. In the case of high-silicon mixtures, the degree of contact between the fuel and oxidizer particles is greater, hence more oxygen atoms will be used to burn the fuel (as assumed in the theoretical model).

**Figure 19:**
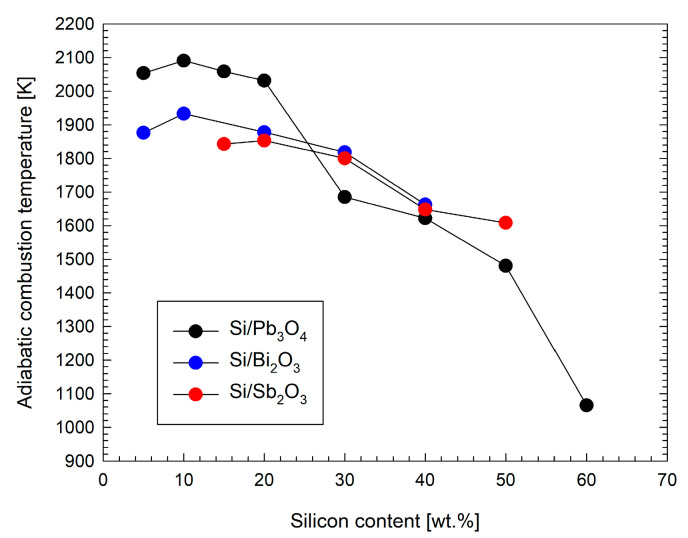
Adiabatic combustion temperature estimated from the experimental heat of combustion as a function of the silicon content.

The measured heat of combustion and assumed composition of the products allowed for the calculation of the adiabatic temperature of combustion (Section 2.7). The temperature dependence of the metal oxide content for the mixtures tested is shown in Figure 19.

The estimated adiabatic combustion temperatures of the mixtures studied are high, especially for mixtures with less silicon. However, the maximum flame-wave temperature determined in [10] for a Si/Sb_2_O_3_ mixture for 20 wt.% Si is close to the adiabatic temperature in Figure 19, while it is 200–250 K lower for higher Si contents. Moreover, the adiabatic combustion temperatures estimated in [10] for mixtures with Si contents from 20 to 50 wt.% are only 100–150 K lower than those shown in Figure 19 for the Si/Sb_2_O_3_ mixture. This comparison indicates that the proposed methodology for determining the adiabatic combustion temperature makes it possible to estimate the combustion temperatures for gasless pyrotechnic mixtures with satisfactory accuracy.

## 4. Conclusions

The series of tests on silicon–metal oxide mixtures described in the article constitutes a practical comparison of the physicochemical properties of potential time-delay compositions. As the research was carried out using explosive detonators, the study of the burn rate allows for the practical application of the results in the design of new delay detonators for civilian use. The research has shown that mixtures based on Pb_3_O_4_, Sb_2_O_3_, or Bi_2_O_3_ are characterized by a wide range of linear burn rates. The highest linear burn rate was obtained for the Si/Pb_3_O_4_ mixture. The presence of two reactions occurring during the combustion of this mixture indicates that it is not completely gasless. An increase in the density of the Si/Sb_2_O_3_ mixture results in an increase in the burn rate, which proves the dominance of the condensed-state reaction system. A similar relationship occurs for the Si/Bi_2_O_3_ mixture, for which, much higher burn rates were recorded comparing with its counterpart containing Sb_2_O_3_, which creates the prospect of using this time-delay mixture as a replacement for mixtures containing lead compounds. The results of the thermal analysis confirmed the conclusions drawn on the basis of the burn rate results—both Si/Sb_2_O_3_ and Si/Bi_2_O_3_ react in a condensed state. Depending on the size of the grains and the burn rate, the components of the mixtures may melt and reactions may occur at the solid–liquid interface, but there is no reaction involving a gaseous intermediary in the form of O_2_.

The ignition temperature of the Si/Sb_2_O_3_ mixture is approximately 600 °C and is almost 300 °C lower than the Si/Bi_2_O_3_ mixture. The analysis of combustion products extends the literature reports to include combustion products of the Si/Bi_2_O_3_ mixture in which bismuth silicates were identified (for a mixture with a positive oxygen balance). These silicates are the reaction product of bismuth oxide, unreacted in the first stage, and the product of the main exothermic reaction—SiO_2_. The analysis of the heat of combustion showed significant discrepancies in the heat of combustion of real samples and theoretical calculations for low fuel contents. A decrease in the oxygen balance contributes to the more ideal combustion of the mixture and, consequently, to obtaining a heat of combustion close to the theoretical one. The Si/Pb_3_O_4_ mixture with a lower silicon content exhibits the highest heat of combustion. The Si/Sb_2_O_3_ mixture shows a slightly lower heat of combustion at a higher silicon content. The mixture based on bismuth oxide demonstrates the lowest heat of combustion among the tested compositions. The calorimetric heat of combustion determined for these mixtures enabled the estimation of the adiabatic combustion temperature using the proposed methodology. The thermochemical results extend existing knowledge of the combustion heat and temperature of the mixtures studied.

The results of this research not only expand the understanding of the combustion process in gasless pyrotechnic mixtures but also assess the suitability of silicon-based mixtures for use in time-delay detonators.

## Figures and Tables

**Table 1:** Theoretical maximum density and particle characteristics of components.

Powder	*TMD* [g/cm^3^]	*D*_[4,3]_ [µm]	*D*_99_ [µm]
Silicon	2.33	7.0	16.0
Antimony(III) oxide	5.20	23.8	49.3
Bismuth(III) oxide	8.90	8.3	15.3
Lead(II,IV) oxide	8.30	8.2	14.1

## Data Availability

The original contributions presented in the study are included in the article; further inquiries can be directed to the corresponding author.
